# A GAN-based fast focusing method for circular SAR images

**DOI:** 10.1016/j.heliyon.2024.e34133

**Published:** 2024-07-04

**Authors:** Bingxuan Li, Yanheng Ma, Lina Chu, Wei Li, Yuanping Shi

**Affiliations:** a Army Engineering University Shijiazhuang Campus, Shijiazhuang, 050000, Hebei, China; b College of Mechanical and Electrical Engineering, Shijiazhuang University, Shijiazhuang, 050011, Hebei, China

**Keywords:** GAN, CSAR, Auto-focus frequency loss, Focus position feature attention

## Abstract

Circular Synthetic Aperture Radar(CSAR) imaging is vulnerable to perturbations in the atmosphere and various other elements that can lead to position offset errors in the antenna's phase center as well as induce motion errors. Traditional phase compensation methods that operate in the time domain, such as Auto-regressive Back-projection (ARBP), typically require computation on a direction-by-direction basis, which can result in the considerable expenditure of time and memory resources. To address these challenges, thispaperintroduces a novel approach for focusing on CSAR images. This method leverages the training of a Generative Adversarial Network (GAN) to directly achieve focus on CSAR sub-aperture images. Additionally, to counteract the network's tendency towards low-frequency preferences, the Auto-focus Frequency Loss (AFFL) is introduced. Moreover, to enhance the accuracy of focus position extraction, the Focus Position Feature Attention (FPFA) is proposed. These innovations, along with a new fusion strategy for the sub-aperture images post-focusing, have been experimentally validated, demonstrating significant improvements in the efficiency and accuracy of CSAR image focusing.

## Highlights

• To solve the problem that deep learning tends to concentrate on learning low-frequency information while ignoring the high-frequency information a self-focus focal frequency loss for SAR images is proposed and named Auto-focus Focal Frequency Loss(AFFL).
• To solve the problem of inaccurate focus position extraction in the image-to-image SAR auto-focusing algorithm, a new focus position feature attention(FPFA) based on SAR images has been proposed. FPFA accurately extracts the spatial location information by coordinate information embedding and coordinate attention generation, and at the same time introduces residual operation to avoid gradient vanishing in the model. The proposed method can balance camouflage target classification's accuracy and real-time performance.
• Proposed a framework of SAR image focusing based on pix2pix, which constructs an adversarial game between the generator and similarity discriminator,The structure of the discriminator has been redesigned according to the SAR focusing task, and the mechanism of the generator was redesigned based on the Unet structure.

## 1 Introduction

Synthetic aperture radar(SAR) [1] is a remote sensing technology based on phased-array radar knowledge, and it generates Earth details images by coherently integrating the motion of the radar platform with the reception of multiple radar echoes. SAR boasts the capability for imaging around the clock and under any weather conditions, which has led to its extensive application and recognized importance across diverse domains [2], including disaster monitoring [3], marine monitoring [4], resource surveying [5], and crop yield estimation [6]. However, inherent restrictions due to physical constraints and imaging parameters can affect the quality of SAR images, resulting in an out-of-focus radar image [7].

CSARrepresents a sophisticated imaging technique that facilitates comprehensive 360° surveillance facilitates comprehensive 360° surveillance of targets [8]. The radar platform is in motion, traversing a circular path with a radius R, centered on the focal point of the environment. It operates at an elevation of h above the ground. The antenna beam is perpetually aligned toward the core of the surveillance area, φ∈[−2π,2π]. To realize its practical utility, CSAR demands the acquisition of high-resolution microwave imagery. In the case of CSAR systems, the aircraft's vibration, compounded by atmospheric perturbations and the impact of wind, can lead to non-linear variations in the antenna phase center (APC). These elements contribute to errors that stem from motion, consequently diminishing the clarity and resolution of the SAR imagery.

With the inception ofSAR, a variety of techniques for automatic SAR image focusing have emerged. These techniques can be broadly classified into two principal groups.

(1) Magnitude-based estimation methods [9], this class of methods contains two types of methods: Map Drift Algorithm(MDA) and metrics-based optimization method. Among them, the sub-aperture method, uses two sub-images to correct second-order phase errors, and multiple sub-images can correct phase errors higher than second-order. Methods based on metric optimization can be further divided into those based on maximizing contrast [10], minimizing entropy [11], and minimizing the norm class methods [12]. These methods treat the problem of auto-focusing as a mathematical optimization problem, which is solved through iterative optimization.
(2) Phase-based estimation methods include the phase gradient method(PGA) and Phase Difference Algorithm(PDA) [13]. The PGA originates from spotlight SARand has been extended to stripmap SAR. This kind of method is capable of correcting phase errors of any order. Similar to the MDA, which uses two looks at an image, PDA can correct second-order phase errors. When employing multiple looks, PDA can correct phase errors higher than the second order. Unlike MDA, which calculates correlation after azimuth compression, the PDA algorithm computes correlation before azimuth.

Due to the specificity of CSAR imaging, the actual process is often considered to be imaged by Back-Projection Algorithm (BP) in the frequency domain. Many scholars have conducted research in this direction, Joshua N proposed an autofocus method on the principle of maximizing image sharpness and solves the phase error correction problem by a simplified HO problem, named Auto-regressive Back-projectionARBP) [14]. Chen introduced sub-aperture image alignment based on ARBP and proposed an image intensity-based CSAR self-focusing algorithm [15]. Aiming at the problem of motion error cavitation, Hu proposed a high-precision motion compensation method, which adopts the optimization technique to estimate the antenna phase center measurement error under the criterion error estimation to correct the antenna phase center and carry out backward projection imaging [16]. It has been proved that metrics-based methods, with good robustness, high focusing quality, and the ability to correct any type of phase error, have been widely used. However, such methods often require thousands of iterations with high computational complexity, making it difficult to meet the demands of real-time processing.

In recent years, remarkable progress has been achieved in the domain of image generation, a development largely fueled by the integration of learning-based models, with a pronounced focus on advanced deep learning methodologies. Within the spectrum of deep learning, two pivotal approaches have come to the forefront: Convolutional Neural Networks (CNNs) and Generative Adversarial Networks (GANs). GANs distinguish themselves through their remarkable capacity to generate photorealistic images. This is accomplished by incrementally mastering the underlying data distribution via the generative network component, in conjunction with conducting adversarial training against a competitive network. This twofold mechanism cultivates a deeper and more sophisticated comprehension of the data, thereby enhancing the quality and realism of the generated images.

However, SAR image auto-focusing has received relatively little research attention, as most existing methods primarily concentrate on optical image super-resolution and SAR image generation. The application of these optical image algorithms to SAR images is often suboptimal, potentially due to inadequate consideration for distinctive SAR image attributes, such as speckle noise. Therefore, specialized research on specific features of SAR images is essential. CNN-based techniques have demonstrated their efficacy in capturing and utilizing the spatial information inherent in SAR images. For example, Shen et al. developed a PolSAR super-resolution framework using a residual CNN, which outperformed conventional techniques in both objective metrics and subjective evaluations [17]. Smith et al. presented an innovative algorithm that integrates a CNN with a visual transformer (ViT), specifically designed for near-field SAR image super-resolution [18]. This hybrid model merges the advantages of CNNs and ViTs, processing both local and global features concurrently, which enhances the detail in the resultant image. Yang et al. improved the resolution of SAR images by using an enhanced nonlocal mean(NLM) to mitigate speckle noise and by refining the loss function based on the structural similarity index [19]. GANs, known for their powerful generative capabilities, are effectively applied to SAR image super-resolution. The NFGAN integrates SAR images denoising with super-resolution reconstruction, effectively reducing noise in the reconstructed images. Chenyi Wang proposed a generative adversarial network with controllable azimuth of SAR target image for the problem that SAR image generation cannot control the azimuth angle, which can generate an accurate SAR image with an azimuth angle between two given SAR image [20]. Shaoyan Du extends the improved Wasserstein loss with gradient penalty into the model to further enhance the diversity and similarity of SAR image generation and validates the proposed algorithm on the MSTAR dataset [21]. Xin Shi proposed an improved GAN (ISAGAN) network with a self-attention mechanism to generate high-fidelity full-azimuth SAR images, which enhances the diversity of the SAR image data [22].

Inspired by image super-resolution and generation algorithms, we prorposeda SAR auto-focus framework for CSAR images. The framework has been carefully designed to achieve auto-focus of the final CSAR images. The main contribution of this paper includes the following:

(1) To solve the problem that deep learning tends to concentrate on learning low-frequency information while ignoring the high-frequency information when facing SAR images, a self-focus focal frequency loss for the task of self-focusing on SAR images is proposed and named as Auto-focus Focal Frequency Loss (AFFL).
(2) To solve the problem of inaccurate focus position extraction that occurs in the image-to-image SAR auto-focusing algorithm, a new focus position feature attention (FPFA) based on SAR images has been proposed. FPFA accurately extracts the spatial location information by coordinate information embedding and coordinate attention generation, and at the same time introduces residual operation to avoid gradient vanishing in the model.
(3) We proposed a framework of SAR image focusing based on pix2pix, which constructs an adversarial game between the generator and similarity discriminator, then learns the distribution of features between the out-of-focus and well-focused SAR images and generates a focused SAR image. Besides, the structure of the discriminator has been redesigned according to the SAR focusing task, and the structureof the generator was redesigned based on the Unet structure.
(4) To improve the accuracy of CSAR sub-aperture alignment, this paper proposed a new alignment strategy for CSAR sub-aperture images by first grouping the sub-aperture images of CSAR, aligning the superposition within each group first, and then superimposing the images of each group at last, which improves the resolution of the final CSAR image.

This paper will be arranged in this way next, the Part II will introduce CSAR imaging and error models, introducing the comomonly used ABP algorithm for CSAR self-focusing and its drawbacks. The Part III reviews and analyzes various fundamental ideas and proposes a new loss for SAR auto-focusing and names it AFFL. The Part IV addressed the prevalent low-frequency bias in network models during the SAR image generation process by employing the Auto-focus Frequency Loss. The Part V introduces the framework of SAR image focusing in details. The Part VI introduces a CSAR subaperture image alignment strategy. The Part VII describes the acquitsition of experimental data and parameter settings, shows the experimental results, and compares them with existing techniques. The Part VIII analyzes the role of AFFL and FPFA in improving the performance of self-focusing and proposes directions for future research, including the potential of GAN networks in compensating for missing orientation information.

## 2 CSAR imaging and error models

**Fig. 1:**
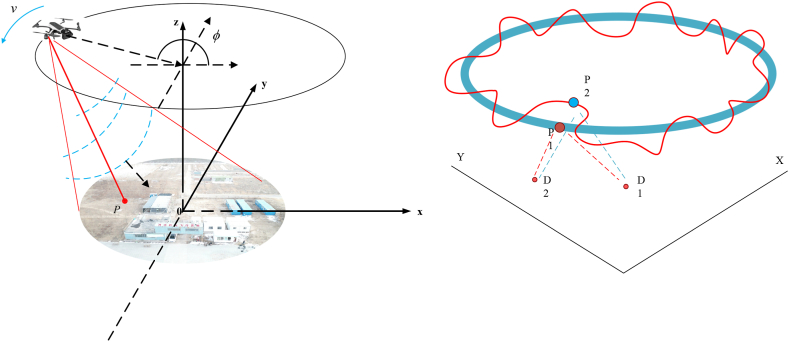
CSAR imaging scene and a schematic of the motion error.

Fig. 1 illustrates the imaging setup for the CSAR system. The radar platform traverses a circular path with radius *R* at an altitude *h* above the scene's central point. The antenna beam is consistently pointed towards the scene's center, with the angle φ ranging from [0,2π]. The coordinates of the radar platform can be derived from the figure as [cos(φ),sin(φ),R+h], In Fig. 1, point *P* is a point target located in the observation plane with coordinates ($\left[x_{p},y_{p},z_{p}\right]$). Consequently, the immediate separation between the radar platform and the target P is as follows:

(1) $$R\left(\varphi ,r_{p}\right)={\left(R\;\cos\;\varphi -x_{p}\right)}^{2}+{\left(R\;\sin\;\varphi -y_{p}\right)}^{2}+{\left(h-z_{p}\right)}^{2}$$

Assuming that the transmitted signal is a Linear Frequency Modulation(LFM) signal, after demodulation and distance compression, the received signal at target P can be expressed as

(2) $$s\left(\tau ,\varphi \right)=\sigma_{p}\times p\left[B\left(\tau -2R\left(\varphi ,\vec{r_{p}}\right)/c_{0}\right)\right]\times \exp\left[-j4\pi f_{c}R\left(\varphi ,\vec{r_{p}}\right)/c_{0}\right]$$

Where $\sigma_{p}$ is the scattering coefficients of the target P, p refers to the distance compression pulse, B is the transmission bandwidth, τ stands for quick time, $c_{0}$ represents the speed of light, $f_{c}$ is the carrier frequency. Assume that r=(x,y,0) refers to the position vector of any grid in the imaging scene, then the value of the CSAR image at the location r can be calculated by

(3) $$z\left(\vec{r}\right)=\int_{0}^{2\pi }S_{rc}\left(R\left(\varphi ,\vec{r_{p}}\right)/c_{0},\varphi \right)\times \exp\left[j4\pi f_{c}R\left(\varphi ,\vec{r_{p}}\right)/c_{0}\right]d\varphi$$

This is the Backward-projection method for integrating along a CSAR aperture. If the BP value of the mth pixel point in the imaging space at the kth slow moment is denoted as $b_{m,k}$, then the complex image value of the mth pixel is

(4) $$z_{m}=\sum_{k=1}^{K}b_{m,k}$$

When phase discrepancies affect the pulses, it is evident that the resulting BPare also compromised in terms of phase integrity just as Eq. (5) shows

(5) $$b_{k}={\tilde{b}}_{k}e^{j\varphi k}$$

However, $\sum {\tilde{b}}_{k}$ can not form a focused image, the purpose of the auto-focus technique is to generate accurate estimations of the phase $\hat{\varphi }=\varphi_{1},\varphi_{2}...\varphi_{k}$. Furthermore, the image that is in focus can be depicted as

(6) $$z_{m}=\sum_{k=1}^{K}{\tilde{b}}_{m,k}e^{-j\tilde{\varphi }k}$$

ABP algorithm considers the sharpness metric $s\left(\overset{⌢}{\varphi }\right)={\sum_{i}v_{i}}^{2}$, and $v_{i}=z_{i}z_{i}^{*}$ refers to the intensity of the i-th pixel. Thus, the final optimization problem can be written as

(7) $${\overset{⌢}{\varphi }}_{k}^{i+1}=\mathrm{argmax}\left(\varphi \right)$$

The BP algorithm is particularly adept at handling CSAR data that is characterized by circular trajectories. In turn, the ABP algorithm excels at rectifying errors that may arise within the BP framework. Nonetheless, when it comes to practical applications, the ABP algorithm is faced with the challenge of having to calculate the error phase for every azimuth direction, a computationally intensive task that can be particularly detrimental to the real-time processing requirements of CSAR imaging.

## 3 Auto-focus frequency loss

### 3.1 Frequency representation of SAR images

In this section, we review and analyze various fundamental ideas associated with the discrete Fourier transform(DFT), illustrate the effects of missing frequencies in synthetic aperture radar (SAR) imagery, and emphasize the advantages of frequency-domain representations pinpointing elusive frequencies. The DFT is a complex valued function that converts a discrete finite signal into its constituent frequencies. SAR images which often represent gray images of the ground's reflective intensity, can be viewed as two-dimensional discrete finite signals with only real numbers. Therefore, to convert a SAR image into its frequency representation, we need to perform a 2D discrete Fourier transform.

(8) $$F\left(u,v\right)=\sum_{x=0}^{M-1}\sum_{y=0}^{N-1}f\left(x,y\right)\cdot e^{-i2\pi \left(\frac{ux}{M}+\frac{vy}{N}\right)}$$

Where the SAR image is M×N,(x,y) is the coordinate of a SAR image pixel in the spatial domain; f(x,y) represents the intensity of reflections in the image. (u,v) denotes the spatial frequency coordinates within the frequency spectrum. F(u,v) denotes the complex frequency value.

(9) $$e^{i\theta }=\cos\;\theta +i\;\sin\;\theta$$

Eq. (9) is known as Euler's formula, e and i represent Euler's number and imaginary unit. By Eq. (9), the natural exponential function in Eq. (8) can be written as

(10) $$e^{i2\pi \left(\frac{ux}{M}+\frac{vy}{N}\right)}=\cos\;2\;\pi \left(\frac{ux}{M}+\frac{vy}{N}\right)-i\;\sin\;2\;\pi \left(\frac{ux}{M}+\frac{vy}{N}\right)$$

**Fig. 2:**
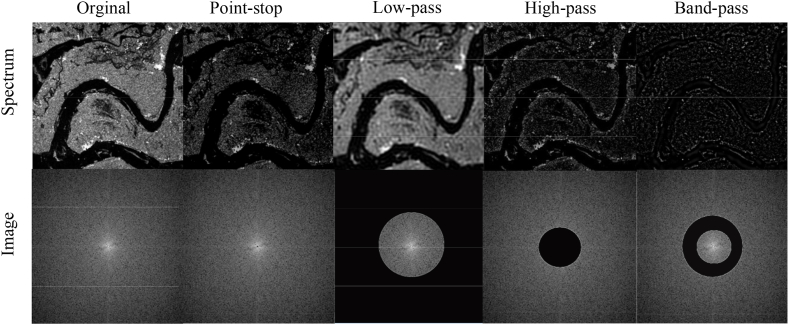
For standard band-limiting operations on the spectrum of a SAR image, the focus is shifted to the origin (low frequencies), and the corresponding image in the spatial domain.

Based on Eq. (8) and Eq. (10), after applying the 2D DFT, the SAR image is decomposed into orthogonal sine and cosine functions, which correspond to the imaginary and real parts of the frequency values. Each of these functions can be seen as a binary function of (x, y), with its angular frequency determined by the spectrum coordinates (u, v). The combination of these sine and cosine functions captures both the horizontal and vertical spatial frequencies present in the image. Spatial frequency is represented by the 2D sinusoidal components within the image. Additionally, the spectrum coordinates (u, v)indicates the directional angle of a spatial frequency—visual, while F(u, v) reflects the image's “response” to that frequency. Owing to the periodic nature of trigonometric functions, the frequency domain representation of an image inherits these periodic characteristics. It can be seen in Eq. (8), that F(u,v) represents the aggregate of a function that iterates over each pixel within the spatial domain, meaning that a specific spatial frequency in the spectrum is influenced by all the pixels in the image. For a clear visual demonstration, we have intentionally removed the central point of the spectrum, which corresponds to the lowest frequency (as shown in Column 2 of Fig. 2), separate low-pass, high-pass, and band-pass operations were performed on SAR images(as shown in Column 3,4,5 of Fig. 2). As seen in the SAR image, the low-pass filter, and the lack of high frequencies in the SAR image, lead to blurring and typical dressing artifacts. In contrast, the lack of low frequencies tends to preserve the edges and boundaries of the SAR images.

**Fig. 3:**
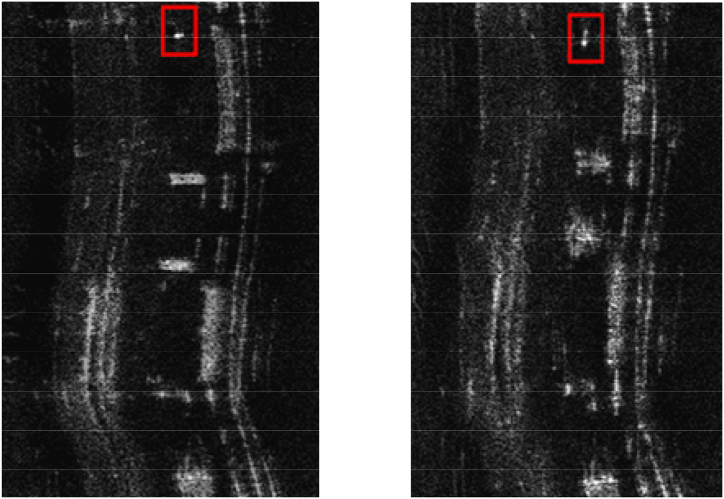
Comparison of SAR images after and before focusing.

For the SAR focusing task, as shown in Fig. 3, the image after focusing has more details compared to the image before focusing, which also means that it has more high-frequency information from the frequency domain. Observably, the degree of focusing a SAR image can be expressed as a loss in different regions of the spectrum. From this, it can be inferred that making up for the missing frequency components could lessen the presence of artifacts and enhance the quality of the SAR images. The analysis presented in this section highlighted the utility of frequency-domain representations in dissecting and accurately localizing various frequency components in an image, especially those that are elusive.

### 3.2 Auto-focus frequency distance

In order to construct a loss function aimed at the missing frequencies, it is essential to employ a differentiable distance metric that captures the disparities between fake and real SAR images within the frequency domain. In this domain, the data entities are the varying spatial frequencies present on the frequency spectrum, which manifest as distinct 2D sinusoidal elements within the image. Returning to Eq. (8), assume that Re(u,v)=x and I(u,v)=y refer to the real and imaginary part of F(u,v), and F(u,v) can be represented as:

(11) F(u,v)=R(u,v)+I(u,v)i=x+yi

For complex frequency value F(u,v), the most important indicators are amplitude and phase, amplitude can be calculated as:

(12) $$\left|F\left(u,v\right)\right|=\sqrt{R{\left(u,v\right)}^{2}+I{\left(u,v\right)}^{2}i}=\sqrt{x^{2}+y^{2}}$$

In the SAR image, amplitude often represents the reflective strength of ground targets. Amplitude also manifests the energy about the degree to which an image reacts to a 2D sinusoidal wave of a particular frequency. The phase of a SAR image can be calculated as:

(13) $$∠F\left(u,v\right)=\arctan\left(\frac{I\left(u,v\right)}{R\left(u,v\right)}\right)=\arctan\left(\frac{y}{x}\right)$$

If we want to establish a new distance, we should consider both phase and amplitude, because they capture different information about the image, and using only phase information or only amplitude information will lead to a failure of the SAR image reconstruction. Assuming that two spatial frequencies, F1 and F2, values are obtained after the image has passed through the DFT. Both spatial frequency values can be expressed in complex form.

(14) $$F_{1}\left(u,v\right)=x_{1}+y_{1}i$$

(15) $$F_{2}\left(u,v\right)=x_{2}+y_{2}i$$

In complex space, the real and imaginary parts correspond to the x-axis and y-axis, respectively, so we can draw a schematic of the two signals in a two-dimensional spatial coordinate system.

**Fig. 4:**
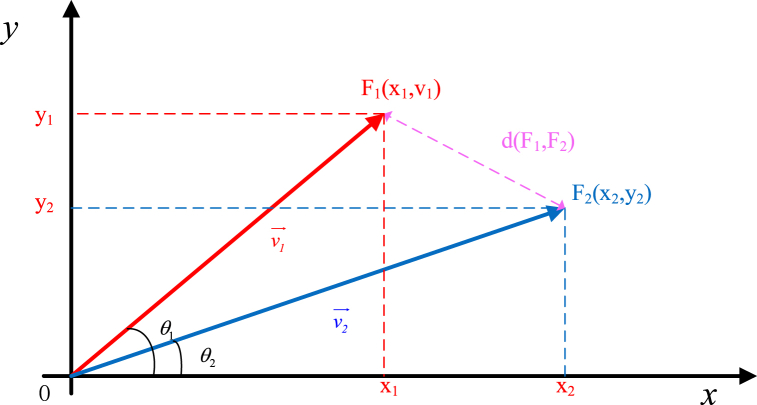
Euclidean distance between two frequency components.

As shown in Fig. 4, we define Euclidean distance as the distance between two frequency components $F_{1}$ and $F_{2}$.It can be seen that the distance $d\left(F_{1},F_{2}\right)$ (purple line) is an adequate measure of the amplitude ${\vec{v}}_{1}$, ${\vec{v}}_{2}$ and phase $\theta_{1}$, $\theta_{2}$ for a single frequency in SAR image, we can calculate the Euclidean distance by Eq. (16), and name it as focus frequency distance.

(16) $$d\left({\vec{v}}_{1},{\vec{v}}_{2}\right)={\left\|{\vec{v}}_{2}-{\vec{v}}_{1}\right\|}_{2}^{2}={\left(y_{2}-y_{1}\right)}^{2}+{\left(x_{2}-x_{1}\right)}^{2}$$

For a SAR image of size M × N, the focus frequency distance can be written as the average value:

(17) $$d\left(F_{1},F_{2}\right)=\frac{1}{MN}{\sum_{u=0}^{M-1}\sum_{v=0}^{N-1}\left|F_{1}\left(u,v\right)-F_{2}\left(u,v\right)\right|}^{2}$$

### 3.3 Dynamic frequency weighting

In the previous section, we defined a new distance to measure the frequency distance in the frequency domain of SAR images, motivating the network to learn the frequency difference between different SAR images. However, it would not make sense to use Eq. (10) directly as a loss function because for each frequency the weights are the same, and the model would still be biased towards learning the easy frequencies due to inherent bias.

In order to force the model to focus the training on the hard frequencies, this section introduces a frequency weight matrix that reduces the emphasis on the less challenging frequencies. This matrix is dynamically adjusted based on a non-uniform distribution of the current loss of each frequency throughout the training process. Each image is assigned to its unique frequency weight matrix. The matrix weight element w(u,v) represents the weight for the spatial frequency at (u,v) and it is defined as:

(18) $$w\left(u,v\right)={\left|F_{1}\left(u,v\right)-F_{2}\left(u,v\right)\right|}^{r}$$

In Eq. (18), r is the scaling factor. We proceed by scaling the matrix values to the interval [0,1], with the value of 1 assigned to the frequency experiencing the greatest loss at present, while the less challenging frequencies receive reduced weight. The gradient concerning the spectrum weight matrix is frozen, meaning it acts solely as a weight for each frequency. By carrying out the Hadamard product—element-wise multiplication—between the spectrum weight matrix and the frequency distance matrix, we obtain the complete structure of the Auto-Focus Frequency Loss (AFFL).

(19) $$L_{AFFL}=\frac{1}{MN}\sum_{u=0}^{M-1}\sum_{v=0}^{N-1}w\left(u,v\right){\left|F_{1}\left(u,v\right)-F_{2}\left(u,v\right)\right|}^{2}$$

## 4 Focus position feature attention

The preceding section addressed the prevalent low-frequency bias in network models during the SAR image generation process by employing the Auto-focus Frequency Loss. However, the SAR image focusing task should somehow be a supervised generative task for GAN networks, because GAN networks need to extract the positional information of the focusing point from the defocused image in order to generate a well-focused image. The steps are very important and directly determine whether the final generated SAR has the characteristics of the original target. In this section, we give an overview of the work on the design of efficient network architectures and attention or non-local models and detail the ideas and derivations used in this paper to design the Focus Position Feature Attention.

### 4.1 Attention mechanisms

Attention mechanisms, which instruct a model on “what” and “where” to focus, have demonstrated their efficacy across a range of computer vision applications, including image classification and image segmentation. A notable example is the Squeeze-and-Excitation Network (SENet) [23], which effectively compressed each 2D feature map to create efficient inter-channel relationships.

Non-local or self-attention networks have gained significant popularity for their adeptness at establishing spatial or channel-specific attention mechanisms. Notable examples such as NLNet, GC-Net, A2Net, SCNe, GSoP-Ne, and CC-Ne leverage non-local strategies to capture a variety of spatial cues. However, these networks are more commonly integrated into larger models and are less feasible for mobile network applications. However, due to the high computational demands of self-attention modules, they are usually integrated into larger models but are not ideal for mobile networks with limited processing capabilities such as GAN network.

### 4.2 Attention mechanisms

A Coordinate Attention block can be considered a computational module designed to augment the representational capacity of features within mobile networks. It accepts input from any intermediate feature tensor $X=\left[x_{1},x_{2}...,x_{c}\right]\in R^{C\times H\times W}$ and produces an output tensor that has been enhanced with richer representations $Y=\left[y_{1},y_{2},...,y_{c}\right]$.

**Fig. 5:**
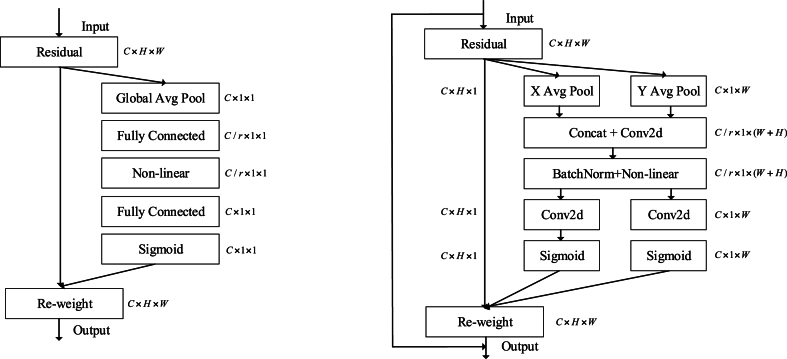
Schematic comparison of the proposed coordinate attention block (a) the classic SE channel attention block (b)Focus Position Feature Attention.

The classic SE channel is shown on the left of Fig. 5, the SE block can be decomposed into two steps, compression, and excitation, for global information embedding and recalibration of channel relations. With input X, the squeeze step of channel c can be expressed as:

(20) $$z_{c}=F_{sq}\left(u_{c}\right)=\frac{1}{H\times W}\sum_{i=1}^{H}\sum_{j=1}^{W}x_{c}\left(i,j\right)$$

In Eq. (20)equation $z_{c}$ is the output associated with the cth channel. The input X comes from a convolutional layer with a fixed kernel size and can therefore be viewed as a collection of local descriptors. The Squeeze operation makes it possible for the model to collect global information.

The second step of SE channel block, excitation, is intended to comprehensively capture channel-wise interdependencies, which can be expressed as:

(21) $$\hat{X}=X\cdot \sigma \left(\hat{z}\right)$$

In the excitation operation, ⋅ refers to channel-wise multiplication, σ refers to the sigmoid function and $\hat{z}$ can be calculated by the $z_{c}$ in the compression, which is formulated as follows:

(22) $$\overset{⌢}{z}=T_{2}\left(\text{Re}LU\left(T_{1}\left(z\right)\right)\right)$$

In Eq. (20), $T_{1}$ and $T_{2}$ represent two linear transformations that are trainable to seize the significance of each channel.

The Squeeze-excitation(SE) block has gained extensive adoption in contemporary mobile networks and has been validated as a crucial element in attaining cutting-edge performance. Nonetheless, the Squeeze-excitationmodule solely accounts for reassigning the significance of each channel through the modeling of inter-channel relationships, neglecting the relevance of spatial relationships, which is however the most important in the SAR auto-focusing task.

Inspired by the idea of SE block, this paper proposes a coordinate attention that encodes both channel relationships and long-range dependencies, a residual module is introduced to accelerate convergence and mitigate gradient vanishing and named it Focus Position Feature Attention(FPFA).

Our Coordinate Attention mechanism effectively captures channel relationships and long-range dependencies by incorporating exact spatial information in a three-step process: the integration of coordinate data, the creation of the attention map through coordinate analysis, and the incorporation of a residual connection for enhanced feature propagation, while being able to mitigate gradient vanishing and accelerate convergence. Fig. 5 illustrates, in its right-hand portion, a schematic representation of the suggested Coordinate Attention module.

Global pooling is commonly used to encode global spatial information for channel attention, but global pooling compresses global spatial information uniformly into channel descriptors, which is not conducive to preserving relative positional relationships in space, which is critical for capturing spatial positional structure in the bokeh image in SAR image focus. To encourage attention blocks to capture long-range interactions in space using precise SAR focus information, we decompose the global set factorization in Eq. 22 into a pair of global set factorizations.

With input X, we employ pooling kernels with two distinct spatial dimensions (H, 1) and (1, W) to capture channel-wise information along the horizontal and vertical axes, respectively. Consequently, the output for the c-th channel at a given height h can be expressed as

(23) $$z_{c}^{h}\left(h\right)=\frac{1}{W}\sum_{0\leq i\leq W}x_{c}\left(h,i\right)$$

and we can calculate the output of the c-th channel at width c as:

(24) $$z_{c}^{h}\left(h\right)=\frac{1}{H}\sum_{0\leq j\leq H}x_{c}\left(j,w\right)$$

The pair of transformations previously referenced merge characteristics across the two dimensions of spatial orientation, resulting in a pair of feature maps that are cognizant of direction. This approach contrasts sharply with the compression operation as in Equation.20 used in channel attention methods, which typically generate a single feature vector. These transformations further empower our attention block to capture long-distance interactions along a single spatial axis, while simultaneously preserving intricate positional details along the other axis. This enhancement bolsters the network's capacity to accurately identify and focus on areas of significance.

The precise focus position information in the SAR image space can be extracted and encoded by Eq. (23) and Eq. (24), to utilize this feature efficiently, We introduced a subsequent transformation, termed the SAR Focus Coordinate Attention Generation. For the aggregated feature mapping extracted by Eq. (23) and Eq. (24), we first connect it by a Concat and then send it to a 1×1 convolutional transform function.

(25) $$f=\delta \left(F_{1}\left(\left[z^{h},z^{w}\right]\right)\right)$$

In the equation, δ refers to the to a non-linear activation function, and [.,.] represents the concatenation operation along the spatial dimension. f can be regarded as an intermediate mapping for encoding spatial information horizontally and vertically. Splitting f into two tensors $f^{h}$ and $f^{w}$ utilizing two additional 1×1 conventional transformation, the result is shown as:

(26) $$g^{h}=\sigma \left(F_{h}\left(f^{h}\right)\right)$$

(27) $$g^{w}=\sigma \left(F_{w}\left(f^{w}\right)\right)$$

The output $g^{h}$ and $g^{w}$ are then expanded and used as attention weights, The networks so designed have significantly increased in width and depth, however, for the original network, simply increasing the depth would have resulted in gradient dispersion or gradient explosion. The solution to this problem is regularization initialization and intermediate regularization layers (Batch Normalization), in which case dozens of layers of the network can be trained. Although the network can be trained by the above method, another problem occurs, the degradation problem, where the number of network layers increases, but the accuracy of the training set is saturated or even decreases. This cannot be interpreted as overfitting and should be characterized by better performance on the training set. The degradation problem shows that deep networks cannot simply be optimized well.

Inspired by residual networks, we have also introduced residual operations in the designed module, connecting inputs and outputs together through direct jump connections allows gradients to propagate more easily, so ultimately the final output of our designed Focus Position Feature Attention can be represented as

(28) $$y_{c}\left(i,j\right)=x_{c}\left(i,j\right)+x_{c}\left(i,j\right)\times g_{c}^{h}\left(i\right)\times g_{c}^{h}\left(j\right)$$

## 5 The framework of SAR image focusing

**Fig. 6:**
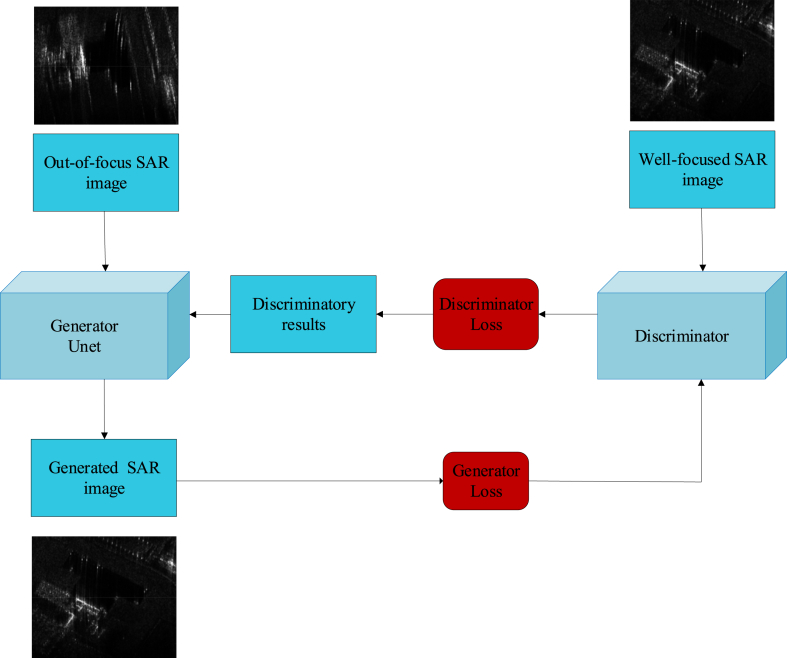
The framework of SAR image focusing.

Fig. 6 shows the framework of SAR image focusing based on Pix2Pix, given two SAR images, $I_{1}$ and $I_{2}$ which refers to an out-of-focus SAR image and a well-focused SAR image. In the Pix2Pix model, $I_{1}$ is the input of the Generator G and generates fake SAR image $I_{g}$. However, in this initial state, the fake SAR image $I_{g}$ has as little information as the real SAR image has. Then, the two images, fake $I_{g}$ and well-focused SAR image $I_{2}$ are inputs to the discriminator D. The discriminator D will determine whether each image is well-focused or not. Through adversarial training, the generator and discriminator are both optimized. Finally, the well-focused SAR image $I_{g}$ will be generated with high quality. The framework for SAR image focusing that we propose leverages a tailored input form and generator-discriminator architecture, enabling a more stable training regime compared to alternative GAN configurations for generating SAR images.

### 5.1 Specific implementation of generator, discrimination

**Fig. 7:**
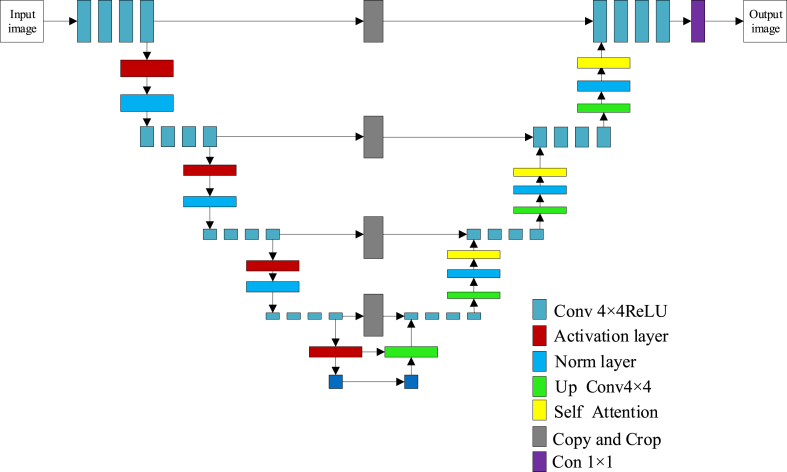
Generator architecture.

**Fig. 8:**
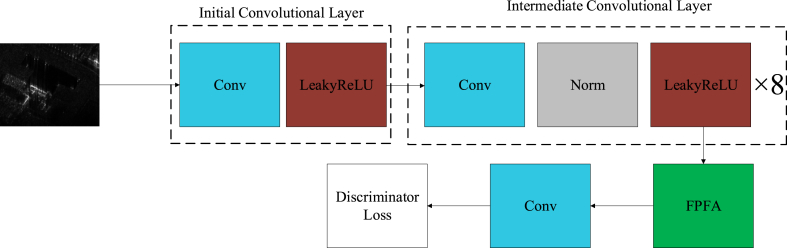
Discriminator structure.

In order to generate a SAR image with well-focused SAR from the input out-of-focus SAR image, the structure of the generator, and the discriminator are designed as shown in Fig. 7 and Fig. 8. The generator in this framework is meticulously crafted, drawing inspiration from the U-net architecture, which is particularly adept at handling image data. It is optimized to receive a SAR image with dimensions of 256 × 256 as input. The image undergoes an initial down sampling process through a layer specifically designed for this purpose, characterized by a kernel size of 4, a stride of 2, and a padding fill value of 1. This setup ensures that the image is effectively reduced in size while retaining essential features. Following the down sampling, a LeakyReLU activation function is applied, which introduces a small negative slope of 0.2 to the output. This activation function is instrumental in mitigating the vanishing gradient problem and allows for a more nuanced representation of negative values within the network. Normalization of the output from the convolutional layer is subsequently carried out by a normalization layer. This step is crucial for maintaining the stability of the learning process and ensuring that the network can learn from a wide range of input values.

The architecture then employs a recursive structure, where each subsequent U-net block incorporates an additional U-net Bloc, thereby constructing the deep and intricate structure that is the hallmark of the U-net model. This design facilitates the feature-encoded paths that are central to the U-net's functionality, enabling the network to learn complex representations of the input data. The feature map is subsequently enlarged through an upsampling layer, which operates with a kernel size of 4, a stride of 2, and a padding value of 1. This upsampling process is critical for increasing the resolution of the feature map, thereby allowing for the reconstruction of a high-quality image. For blocks that are not part of the innermost layers, the ReLU activation function is reapplied in conjunction with the normalization layer. This combination ensures that the non-linear transformations necessary for feature extraction are effectively captured. To enhance the capabilities of the U-net model, a self-attention module has been integrated into the architecture. This module is designed to improve the network's ability to focus on relevant parts of the input data, thereby facilitating more accurate and efficient feature extraction and image reconstruction. The inclusion of the self-attention mechanism represents a significant extension to the traditional U-net model, endowing the generator with a heightened level of sophistication and adaptability.

The discriminator's architecture is thoughtfully structured, as delineated in Fig. 8, to ensure robust feature extraction and image discrimination capabilities. It is composed of several key components, including the initial convolutional layer, the intermediate convolutional layers, the Focus Position Feature Attention (FPFA) module, and the final convolutional layer.

The initial convolutional layer is a pivotal element that sets the stage for subsequent processing. It employs a Conv2d layer to handle the input image, configured with a kernel size of 4, a stride of 2, and a padding of 1. This configuration allows for an effective reduction in the spatial dimensions of the image while simultaneously capturing local features. After this, a leakyReLU activation function is applied, which incorporates a negative slope of 0.2 to add depth to the non-linear transformations and to aid in preventing issues related to vanishing gradients.

Following the initial layer, a sequence of intermediate convolutional layers is introduced. Each layer in this series progressively increases the number of filters, thereby expanding the model's capacity to detect a diverse range of features within the input image. These layers consistently utilize the Conv2d operation, which is then followed by a normalization layer to ensure that the activations are regularized, and capped with an LeakyReLU activation layer to maintain non-linearity and facilitate effective learning. The FPFA module is strategically integrated after the intermediate layers to sharpen the discriminator's focus on the spatial relationships within the image. This attention mechanism is instrumental in identifying and emphasizing the most salient features and positions within the input data. Post the FPFA, a subsequent Conv2d layer is employed to condense the feature map into a single channel. This dimensionality reduction generates an output map that provides a binary probability assessment for each segment of the input image, indicating the likelihood of its authenticity. In this final convolutional operation, a kernel size of 4 is used with a stride of 1 to preserve the spatial integrity of the input while performing this critical evaluation. The discriminator's design thus encapsulates a comprehensive and nuanced approach to image analysis, ensuring a high degree of accuracy and reliability in the SAR image focusing framework.

### 5.2 Loss function

The traditional GAN loss function (objective function) is expressed as

(29) $$L_{GAN}\left(f_{G},f_{D}\right)=\min_{G}\max_{D}E\left(\ln\;f_{D}\left(x\right)\right)+E(\ln\left(1-f_{D}\left[f_{G}\left(z\right)\right]\right)$$

where x is the image data; z is the random noise; E refers to the expected value; $f_{D}$ refers to the output of the discriminator D and $f_{G}$ refers to the output of the generator G. The classical GAN model generates content determined by the parameter $f_{G}$ $ and random noise z, and has no control over the generated content. To exert precise control over the generator's output, the concept of conditional information, denoted as y, is introduced. In the context of the Pix2Pix model, y represents data from another image domain that is aligned with the target domain m. This model utilizes y as a reference to ensure that the generated content is not only novel but also relevant and specific to the desired output domain. Within the Pix2Pix model, y serves as a crucial element that guides the generator in producing a cross-modal feature. The generator takes in both the random noise z and the conditional data y, combining these inputs to synthesize an output that bridges the gap between the input and output domains. This process allows the generator to create content that is not only stochastically diverse but also coherent with the target domain. Adding the conditional information y, the loss function $L_{pix2pix}$ for Pix2Pix has the expression as:

(30) $$L_{pix2pix}\left(f_{G},f_{D}\right)=\min_{G}\max_{D}E\left(\ln\;f_{D}\left(x\right)\right)+E(\ln\left(1-f_{D}\left[f_{G}\left(z\right)\right]\right)$$

Typically, incorporating an L1 regularization term in the loss function can lead to the sparsification of model parameters, thereby enhancing generalization capabilities and aiding in the generation of clearer data. The L1 regularization term in the loss function, denoted as L1, is given by

(31) $$L_{L1}\left(f_{G}\right)=E\left[{\left\|y-f_{G}\left(m,z\right)\right\|}_{1}\right]$$

On this basis, in order to guide the model to better focus on the high-frequency information in the image, the Auto-focus Frequency Loss mentioned before is added, and the finally loss can be calculated as

(32) $$L_{G}=L_{GAN}+L_{L1}+0.5L_{AFFL}$$

The discriminator loss employed in our approach is formulated as follows:

(33) $$L_{D}=-E\left[\min\left(0,-1+D\left(m,y\right)\right)\right]-E\left[\min\left(0,-1-D\left(G\left(z\right),y\right)\right)\right]$$

## 6 CSAR subaperture image alignment strategy

**Fig. 9:**
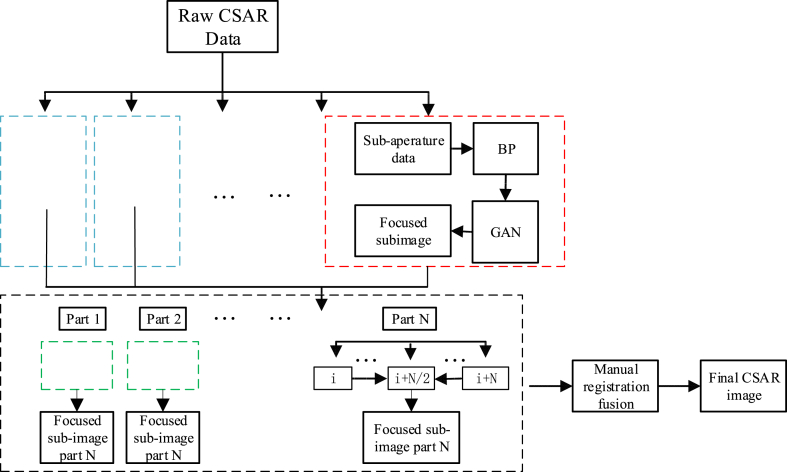
CSAR sub-aperture alignment method.

The final step after obtaining all CSAR sub-images is to precisely combine them together. In CSAR imaging, the variance of the observing trajectory and the sampling frequency can cause position errors for the pixels in the images, resulting in geometric distortions such as offset and scaling. Additionally, the estimated phase errors in individual CSAR data may be inconsecutive among CSAR, leading to different azimuth offsets of the sub-images. Therefore, an image registration method is required to combine the sub-images. Chen proposed a method using the first sub-image as the global reference image for the other sub-images. However, as the azimuth angle of the CSAR keeps changing, the scatter characteristics of the target scene vary greatly, making it difficult to align with the first sub-image. To solve this problem we propose a segmented CSAR sub-aperture image synthesis method and the specific flow is shown in Fig. 9. Assuming CSAR data is divided into M sub-apertures and partitioned into N parts. Each part consists of sub-apertures [i, i + N/2 … i + N]. Choose i + N/2 as a reference sub-aperture, registering all sub-aperture images in each part with respect to the reference sub-aperture by DLSC\cite{7862207}. The accumulation results for each part are shown in equation (27). The final CSAR image is obtained by manually registering different parts and performing incoherent addition.

(33) $$A=\sum_{i}^{i+N}S_{i}/N$$

## 7 Experiments and results

### 7.1 Dataset

The experiments used in this paper are colleted by an air borne CSAR data acquisition experiment, which was mounted on an unmanned aerial vehicle(UAV) in the Chang'an Street District of Shijiazhuang. The CSAR system parameters are presented in Table 1.

**Table 1:** CSAR data parameters settings.

Parameter	Value	Parameter	Value
TX frequency	9.6 GHz	Flight radius	600 m
Bandwidth	0.75 GHz	PRF	333.3 Hz
velocity	7 m/s	Slant-range	500–1000 m

**Fig. 10:**
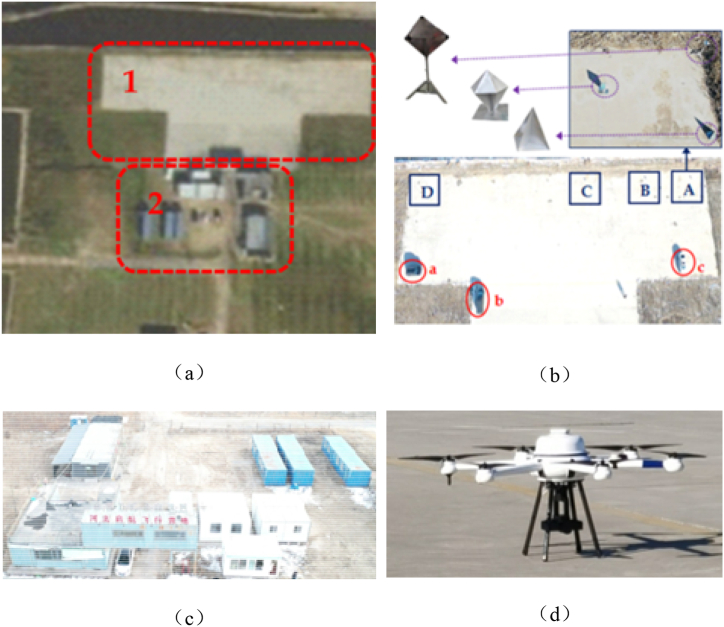
Schematic diagram of the experimental site's target setup and carriers.

Fig. 10 shows the experimental scene and vehicle, the region marked in as Area 1 comprises of a “T"-shaped cement ground that houses three vehicles, namely A, B and C, along with four reflectors, A,B,C and D. Three reflectors are divider into groups and each group consists of a tetrahedral angle reflector, a hexahedral angle reflector, and a dihedral angle reflector. The second area consists of a house constructed form separate containers, as depicted in Figure (c). Figure (d) shows the unmanned aerial vehicle (UAV) carrying the radar. To enhance the diversity of the data, the data was acquired using different elevation angles and flight radii, the specific parameters can be seen in Table 2.

**Table 2:** CSAR data parameters settings.

Flight radius/m	Altitude of flight/m	Antenna phase Center angle	center-slope distance/m
600	450	53.1301	600
600	350	59.7436	694.6222
600	275	65.3764	660.0189
700	500	54.4623	860.2325
640	425	56.4134	768.2610
640	425	56.4134	768.2610
580	350	58.8912	677.4216
520	275	62.1280	588.2389

**Fig. 11:**
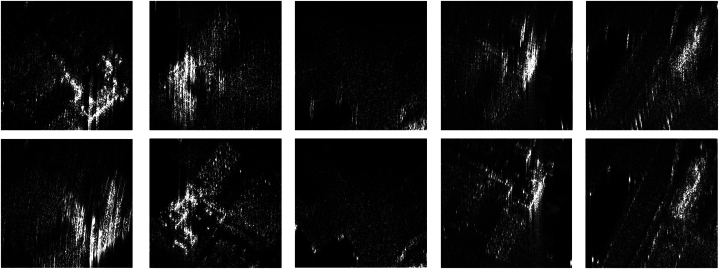
Schematic diagram of SAR image pairs of the dataset.

To generate the dataset for this paper, the CSAR data in Table 2 were imaged using the BP algorithm, followed by self-focusing using the ABP algorithm, and sequentially combined in pairs to form a set of inputs as Fig. 11 shows, based on the collected data we produced a total of 8460 corresponding SAR images to form the dataset. In the experiments in this paper, we use the Adam optimizer to train where beta_1 = 0.45, beta_2 = 0.999, the total iteration number is 30000, and the batch size is 64. The learning rate for the discriminator is 0.0004 and the learning rate for the generator is 0.00001. The duration of instance noise is 30000 iterations. The proposed methods are tested and evaluated on a computer with Inter Core I7–12700 KF at 3.6 GHz CPU, and GeForce GTX 4070GPU with 12 GB memories. The proposed method is implemented using the open-source Pytorch framework. Fig. 11 shows some of the sub-apertures of the dataset produced in this paper.

### 7.2 Result and analyze

In this section, following the presentation of the dataset setup for assessing the generative performance, we will evaluate the generative capacity of the proposed network, which is designed to generate well-focused SAR images. We evaluate the superiority of the proposed network form two perspectives. Initially, a comparison will be made between the advantages of the proposed network and other self-focusing methods. Subsequently, an ablation study will be conducted to confirm the enhancement of self-focusing effects brought about by the proposed AFFL and FPFA components.

#### 7.2.1 Comparison of the proposed method with conventional self-focusing methods

To demonstrate the superiority of the GAN network-based approach for SAR self-focusing, the PGA, and MEA algorithms are used in this paper for the test data to be imaged.

**Fig. 12:**
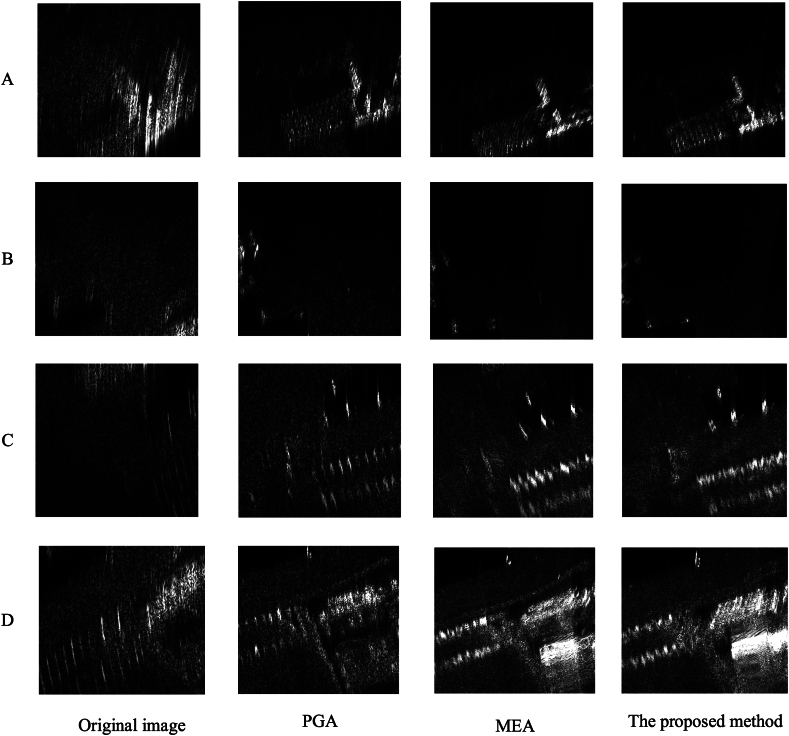
Comparison of imaging results(*a*)Original image (b)PGA (c)MEA (d)The proposed method.

As shown in Fig. 12, we can observe that compared to the originally defocused CSAR images, both PGA, MEA, and the method proposed in this paper exhibit certain focusing effects. The focusing effect of PGA is very limited, and the ghosting phenomenon of the scene targets is still very severe. This is primarily because PGA often assumes a straight flight trajectory when applying a window. For CSAR data, the PGA algorithm can be used for a small range of SAR auto-focus. However, when the azimuth direction varies significantly, the focusing effect of PGA will decrease very noticeably.

Meanwhile, compared with MEA, the proposed method in this paper performs better in details, thanks to the AFFL and FPFA proposed in this paper, and in order to better measure the differences between the methods.

**Fig. 13:**
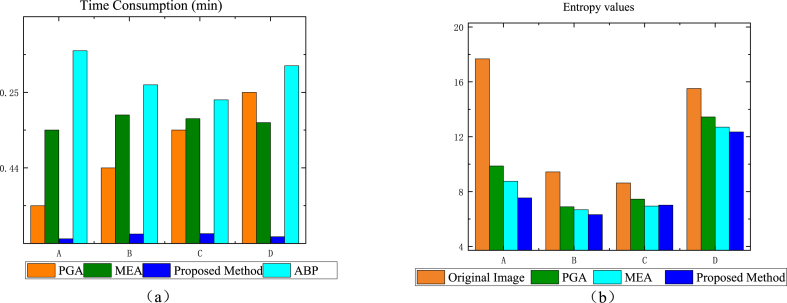
Comparison of time consumption and image entropy value of this paper's method with traditional methods. (a)Time Consumption(min) (b)Entropy values.

In order to further assess the effectiveness of the auto-focus capabilities of the algorithm introduced in this study, the entropy metric was employed as a numerical measure of image sharpness. Typically, an image with greater blurriness corresponds to increased uncertainty, which is reflected by a higher entropy value. The result is shown in Fig. 13. For Group A data, the entropy value of the proposed method in this paper, 7.54, is much lower than that of PGA, 9.87, and that of MEA, 8.76; for Group B data, there is not much difference among the three groups of methods, with the method of this paper slightly ahead of that of PGA, 6.9, and that of MEA, 6.68, with 6.32; for Group C data, the MEA performs the best, with 6.94, followed by that of this paper's method, 7.01, and 7.45; for the Group D data, this paper's method leads with 12.35 over PGA's 13.44 and MEA's 12.7.

To better illustrate the superiority of the method in this paper, we compare the processing time and the entropy of the three methods for a single image, as shown in Fig. 13. Among several conventional SAR auto-focusing, the method in this paper achieves the lowest time consumption. This is mainly because auto-focusing algorithms for CSAR are generally metrics -based optimization methods, regardless of whether they are intensity-optimal or entropy-optimal, which generally need to be optimized directional, whereas methods based on GAN generation can be processed directly from the image, which greatly saves computational resources and time.

#### 7.2.2 Ablation experiments for AFFL and FPFA

**Fig. 14:**
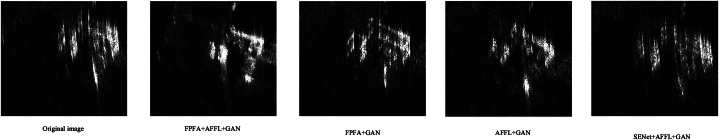
Comparison of ablation experiments in FPFA and AFFL.

To better verify our contributions, this section conduct ablation studies on the validation we created and discuss the details below. We conduct ablation study by replacing the FPFA and AFFL with other types of alternatives modules. To be specific, we implemented three alternatives. One is to directly use the full connections instead of FPFA while adding AFFL to the loss function, the other is to incorporate the FPFA mechanism, but the loss function is replaced with a regular GAN, the third method is to use the SENet module in place of the FPFA module. We reported the quantitative comparison in Table 3 and visualize proposals from different approaches in Fig. 14.

**Table 3:** Ablation experiments for AFFL and FPFA.

Method	Entropy value	Contrast Value	Time Comsume(s)
Original image	10.9441	4.3215	
FPFA + AFFL + GAN	9.5800	5.9154	27.32
FPFA + GAN	9.7832	5.8532	16.21
AFFL + GAN	9.7163	5.6637	12.32
SENet + AFFL + GAN	9.6987	5.7432	32.17

From Table 3 and Fig. 14, it can be observed that the GAN-based framework can effectively learn the focusing patterns of SAR images and perform focusing on the images. Meanwhile, in terms of the entropy value and contrast value of the image after focusing, the method proposed in this paper has the best results. In time comsume, the focusing time of the proposed method is 27.32s, which is faster than the 32.17s of SEnet + AFFL + GAN, and slightly slower than the other combinations of methods, which is mainly due to the small amount of computations of the other methods. In terms of focusing effect, compared to other methods, the proposed method in this paper concentrates the energy more and the outline of the building is more clear than the other method. Also the comparison graph can show that FPFA can effectively extract the position information of the focusing point than SEnet, so as to realize the better focusing effect of the SAR image.

### 7.3 CSAR sub-aperture image incoherent superposition

Once the CSAR sub-aperture images have been focused, it is necessary to operate non-coherent stacking of these sub-aperture images to amalgamate the comprehensive spatial information of the scene effectively.

**Fig. 15:**
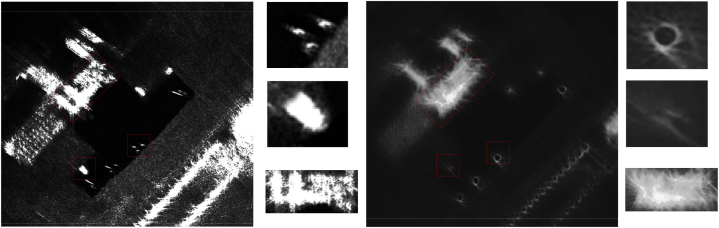
Comparison of circumferential CSAR imaging results.

Fig. 15 presents a comparison between the CSAR image processed through the framework of this paper and the original CSAR image. The processed image exhibits a marked enhancement in clarity, with the three corner reflectors now distinctly identifiable, the outlines of the vehicles more prominently defined, and the houses discernible in their structural form. Additionally, this study computes the entropy values of the two images, yielding 13.4 for the processed image and 17.8 for the original. These entropy values further substantiate the efficacy of the methodology proposed within this paper.

## 8 Discussion

The framework introduced in the paper incorporates two key innovations: the Auto-focus Frequency Loss (AFFL) and the Focus Position Feature Attention (FPFA). The AFFL is designed to counteract the network's natural tendency to focus on low-frequency information, which is a common issue in deep learning when dealing with SAR images. By enhancing the low-frequency response, the AFFL ensures that the generated images retain the necessary details for accurate interpretation.

The FPFA module is another crucial component that addresses the challenge of accurately extracting focus position information. By embedding coordinate information and generating attention through residual operations, FPFA prevents gradient vanishing and ensures that the model can accurately identify and focus on areas of significance within the SAR images.

The experimental validation of the proposed method demonstrates a significant improvement in both the speed and precision of autofocusing. However, it is observed that some sub-aperture images in CSAR remained suboptimal even after autofocusing, leading to the omission of certain angular scene information. This observation opens new avenues for future research, where the GAN network could be utilized to compensate for the missing azimuthal information. The potential of the GAN network to fill in the gaps left by traditional autofocusing algorithms is an exciting direction for future work. This could involve the development of more sophisticated loss functions and attention mechanisms that can better capture the complex features of SAR images. Additionally, the integration of the proposed method with other advanced machine learning techniques could further enhance its performance and applicability.

In conclusion, this paper presents a new approach to CSAR image autofocusing using GANs. The introduction of the AFFL and FPFA module addresses critical challenges in the field, and the experimental results validate the effectiveness of the proposed method. The work of this study, which demonstrate the efftiveness of the GAN-based autofocusing method for SAR images, contributes significantly to the field of remote sensing, our approach not only improves the quality of SAR imagery but also offers a more efficient processing time compared to traditional methods. This advancement has imlications for real-time monitoring applicaions, where rapid and accurate image analysis is crucial. Howere, our study has some limitations, including the reliance on specific types of SAR data and the potential for overfitting to the training dataset. Future research should address these limitations by exploring the method's applicability to diverse SAR data and incorporating robust regularization techniques. Additionally, the integration of our method with other image processing technologies presents an exciting avenue for enhancing multimodal remote sensing capabilities.

## Funding

This research did not receive any specific grant from funding agencies in the public, commercial, or not-for-profit sectors.

## Data statement

The measured data belongs to the Army Engineering University and is not suitable for publication due to copyright protection.

## CRediT authorship contribution statement

**Bingxuan Li:** Writing – review & editing, Writing – original draft, Data curation, Conceptualization. **Yanheng Ma:** Writing – review & editing, Writing – original draft, Data curation, Conceptualization. **Lina Chu:** Methodology, Investigation, Formal analysis. **Wei Li:** Methodology, Investigation, Formal analysis. **Yuanping Shi:** Methodology, Investigation, Formal analysis.

## Declaration of competing interest

The authors declare that they have no known competing financial interests or personal relationships that could have appeared to influence the work reported in this paper.
